# Fabrication and Oxidation Resistance of Metallic Ta-Reinforced High-Entropy (Ti,Zr,Hf,Nb,Ta)B_2_ Ceramics

**DOI:** 10.3390/ma18194642

**Published:** 2025-10-09

**Authors:** Bowen Yuan, Qilong Guo, Hao Ying, Liang Hua, Ziqiu Shi, Shengcai Yang, Jing Wang, Xiufang Wang

**Affiliations:** 1 School of Civil Engineering, Northwest Minzu University, Lanzhou 730124, China; 2 Key Laboratory of New Building Materials and Building Energy Efficiency of Gansu Province, Lanzhou 730124, China

**Keywords:** high-entropy boride, tantalum, fracture toughness, toughening mechanism, oxidation resistance

## Abstract

High-entropy boride (HEB) ceramics combine ultra-high melting points, superior hardness, and compositional tunability, enabling service in extreme environments; however, difficult densification and limited fracture toughness still constrain their aerospace applications. In this study, metallic Ta was introduced into high-entropy (Ti_0.2_Zr_0.2_Hf_0.2_Nb_0.2_Ta_0.2_)B_2_ as both a sintering aid and a toughening phase. Bulk HEB-Ta composites were fabricated by spark plasma sintering to investigate the effect of Ta content on densification behavior, microstructure, mechanical properties, and high-temperature oxidation resistance. The results show that an appropriate amount of Ta markedly promotes densification; at 10 vol% Ta, the open porosity reaches a minimum of 0.15%. Hardness and fracture toughness exhibit an increase-then-decrease trend with Ta content, attaining maxima at 15 vol% Ta (20.79 ± 0.17 GPa and 4.31 ± 0.12 MPa·, respectively). During oxidation at 800–1400 °C, the extent of oxidation increases with temperature, yet the composite with 10 vol% Ta shows the best oxidation resistance. This improvement arises from the formation of a viscous, protective Ta_2_O_5_-B_2_O_3_ glassy layer that effectively suppresses oxygen diffusion and enhances high-temperature stability. Overall, incorporating metallic Ta is an effective route to improve the manufacturability and service durability of HEB ceramics, providing a composition guideline and a mechanistic basis for simultaneously enhancing densification, toughness, and oxidation resistance.

## 1. Introduction

Rapid advances in aerospace, nuclear engineering, armor protection, and mechanical metallurgy are imposing ever more stringent requirements on the stable service of ultra-high-temperature materials (UHTMs) under extreme environments [1,2,3]. To meet the special demands of high-temperature exposure and heavy-load operation in such conditions, and building on conventional UHTC systems [4,5,6], the “high-entropy” design paradigm from alloy science has been adopted [7,8,9] to develop a family of novel transition-metal, non-oxide high-entropy UHTCs. Among these, high-entropy borides [10,11], carbides [12,13], and nitrides [14,15] have emerged as major research foci. High-entropy boride ceramics combine ultra-high melting points, exceptional hardness, good oxidation and corrosion resistance, excellent high-temperature stability, and compositional tunability, conferring significant application potential in extreme environments such as aerospace, nuclear reactors, and high-speed machining [7].

High-entropy boride ceramics adopt the AlB_2_-type hexagonal structure [16], in which both B-B and metal-B bonds exhibit strong covalency. This bonding confers ultrahigh melting points while simultaneously making sintering densification difficult. Gild et al. [10] sintered five commercial diboride powders at 2000 °C and obtained samples with relative densities of only 92–93%, which falls short of the requirements for ultra-high-temperature service. To address the densification challenge, progress has been achieved by preparing high-quality powders, adding sintering aids, and employing advanced consolidation techniques, for example: (1) alternative sintering routes such as spark plasma sintering (SPS) [17,18], self-propagating high-temperature synthesis–spark plasma sintering (SHS-SPS) [19,20], hot pressed sintering (HPS) [21], oscillatory pressure sintering (OPS) [22,23], and high-pressure sintering [24]; and (2) the addition of second-phase sintering aids [16], e.g., C [25] and SiC [26], which can react with the matrix to form new compounds or solid solutions, optimize grain boundary chemistry and structure, and enhance intergranular bonding. Meanwhile, the strategy of introducing metallic additives into high-entropy ceramics as sintering aids has also been explored. For instance, Liang Xu et al. [27] used Co to successfully fabricate fully dense, fine-grained high-entropy (Hf,Zr,Ta,Nb,Ti)B_2_ ceramics, and Huang et al. [28] produced dense high-entropy (Hf,Mo,Ta,Nb,Ti)B_2_ ceramics by introducing Ni under pressureless sintering at 1800 °C.

Another critical limitation of high-entropy boride ceramics is their low fracture toughness, typically 2–4 MPa·m^1/2^, which severely restricts widespread deployment under extreme conditions [29,30,31]. Common toughening strategies—such as particle dispersion toughening [26], transformation toughening [32], self-toughening [33], and whisker toughening [34]—rely on pull-out/bridging of added phases and crack deflection at phase interfaces; however, the improvements remain limited, with toughness generally hovering around 4–8 MPa·m^1/2^. Building on the idea of tailoring the composition, size, and volume fraction of the toughening phase, optimizing its spatial architecture can further increase toughness substantially, reaching 14.22 MPa·m^1/2^; nevertheless, the enhancement is strongly direction-dependent, which constrains practical applicability [35].

Notably, metal particles also enhance the toughening effect, and the improvement in fracture toughness is multi-directional, not limited to specific orientations. By maximally suppressing the volumetric effects of matrix defects and activating toughening mechanisms such as crack deflection, crack bridging, and crack branching, the fracture toughness of ceramics is increased. Recently, researchers have successfully fabricated cermets composed of high-entropy carbide ceramics and refractory metals (Co, Ni) [36,37,38]. Among them, the co-addition of Ni and Co gives the best toughening effect, with the fracture toughness reaching 14 MPa·m^1/2^. These studies indicate that the addition of metals can improve not only sintering densification but also fracture toughness. Among such metals, metallic Ta [39] has a high melting point (3020 °C), good toughness, excellent high-temperature strength, creep properties, and thermal shock resistance, as well as good oxidation resistance [40,41,42,43], and its oxidation products are resistant to high temperatures [44,45,46].

Accordingly, metallic Ta was employed as a toughening phase and sintering aid and uniformly mixed with (Ti_0.2_Zr_0.2_Hf_0.2_Nb_0.2_Ta_0.2_)B_2_ (HEB) powders. HEB-Ta composites were fabricated by SPS. We investigated the effects of Ta content on the composite ceramic’s phase composition, microstructure, mechanical properties, and high-temperature oxidation resistance; examined how Ta content influences the fracture process and crack propagation mode; elucidated the toughening mechanism; and clarified the impact of Ta content on high-temperature oxidation performance.

## 2. Experimental Details

### 2.1. Preparation

Raw materials: TiO_2_ (1–3 μm, purity ≥ 99.90%, Qinhuangdao Yinuo High-tech Materials Development Co., Ltd., Qinhuangdao, China), ZrO_2_ (1–3 μm, purity ≥ 99.90%, Qinhuangdao Yinuo High-tech Materials Development Co., Ltd.), HfO_2_ (−0.3 μm, purity ≥ 99.95%, Beijing Fangdexing Technology Co., Ltd., Beijing, China), Nb_2_O_5_ (5–10 μm, purity ≥ 99.90%, Qinhuangdao Yinuo High-tech Materials Development Co., Ltd.), Ta_2_O_5_ (1–3 μm, purity ≥ 99.90%, Shanghai Naio Nanotechnology Co., Ltd., Shanghai, China), B_4_C (0.5 μm, purity ≥ 99.90%, Shanghai Naio Nanotechnology Co., Ltd.), C (−2.0 μm, purity ≥ 99.99%). Ta powder, diameter (−45 μm, purity ≥ 99.90%, Qinhuangdao Yinuo High-tech Material Development Co., Ltd.).

HEB powder was synthesized as follows [47]: TiO_2_, ZrO_2_, HfO_2_, Nb_2_O_5_, and Ta_2_O_5_ were weighed at equimolar metal-atom ratios; B_4_C was added at 20 wt% in excess of the theoretical stoichiometric requirement. The mixed powders, with an additional 6 wt% carbon serving as a reductant, were reacted under vacuum in a furnace (ZT-50-20Y, Shanghai Chenhua, Shanghai, China), heated at 10 °C·min^−1^ to 1700 °C and held isothermally for 1 h, yielding single-phase, high-purity HEB powder.

HEB powder was homogeneously mixed with metallic Ta powder at 0, 5, 10, 15, and 20 vol%, then loaded into a graphite die (25 mm inner diameter) and consolidated by SPS. The heating rate was 100 °C·min^−1^, the uniaxial pressure was 30 MPa, and the dwell at the final temperature was 10 min. To determine the optimal sintering temperature, the composition containing 20 vol% Ta was first sintered over 1800–2000 °C. The specimens containing 0–15 vol% Ta were subsequently sintered at the optimized temperature. The sintered composite ceramics were designated HEB-0Ta, HEB-5Ta, HEB-10Ta, HEB-15Ta, and HEB-20Ta.

### 2.2. Characterization and Measurement

Phase composition was determined by X-ray diffraction (XRD; X’Pert PRO, Philips, Amsterdam, The Netherlands). Microstructure was examined using scanning electron microscopy (SEM; EVO 18, Carl Zeiss, Cambourne, UK). Local microstructure and composition were characterized using a 200 kV scanning transmission electron microscope (STEM; Talos F200, Thermo Scientific, Waltham, MA, USA). Both electron microscopes were equipped with energy-dispersive X-ray spectroscopy (EDS) for elemental mapping. Open porosity was measured using Archimedes’ method. Vickers hardness was measured using a microhardness tester (HVS-30Z, Shanghai Taiming Optical Instrument Co., Ltd., Shanghai, China) at a load of 49 N with a 15 s dwell.

Composite ceramic rectangular bars (22 × 2 × 4 mm) were prepared by wire-saw cutting for single-edge notched beam (SENB) testing to determine fracture toughness. (support span 16 mm; crosshead rate 0.05 mm·min^−1^), with *K_IC_* calculated as:

(1) $$K_{IC}=\frac{F\times L}{B\times W^{\frac{3}{2}}}\times f(\frac{a}{w})$$

In Equation (1), *K_IC_* denotes the fracture toughness (MPa·m^1/2^), *P* is the applied load (N), *S* is the support span (mm), *B* is the specimen thickness (mm), *W* is the specimen width (mm), and *a* is the notch depth, set to *a*/*w* = 0.4–0.5. The notch depth was <0.2 mm. *f*(*a*/*w*) is the dimensionless geometry factor, given by:

(2) $$f(\frac{a}{w})=\frac{3\;{\left(\frac{a}{w}\right)}^{\frac{1}{2}}\times [1.99-(\frac{a}{w})\times (1-\frac{a}{w})\times (2.15-3.93\times (\frac{a}{w})+2.7\times {\left(\frac{a}{w}\right)}^{2})]}{2\times (1+2\frac{a}{w})\times {\left(1-\frac{a}{w}\right)}^{\frac{3}{2}}}$$

Oxidation resistance was evaluated by a calcination method. Rectangular prisms (5 × 3 × 4 mm) were machined and ultrasonically cleaned. After drying, the initial mass (*W*_0_), was measured on an analytical balance, and the total geometric surface area (*A*) was calculated from the specimen dimensions. The specimens were placed on a grooved refractory setter and calcined in ambient air in a box furnace (KSL-1800X, Hefei Kejing, Hefei, China). After cooling, the final mass (*W*_1_) was measured, and the mass gain per unit area (∆m/A) was determined. A larger mass gain indicates poorer oxidation resistance. The mass gain per unit area was calculated as:

(3) $$\Delta W=\frac{W_{1}-W_{0}}{A}$$

In Equation (3), ∆*W* denotes the mass gain per unit area (mg/cm^2^), *W*_0_ and *W*_1_ are the specimen masses before and after oxidation (mg), respectively; and *A* is the total pre-oxidation surface area of the composite ceramic.

## 3. Results and Discussion

**Figure 1:**
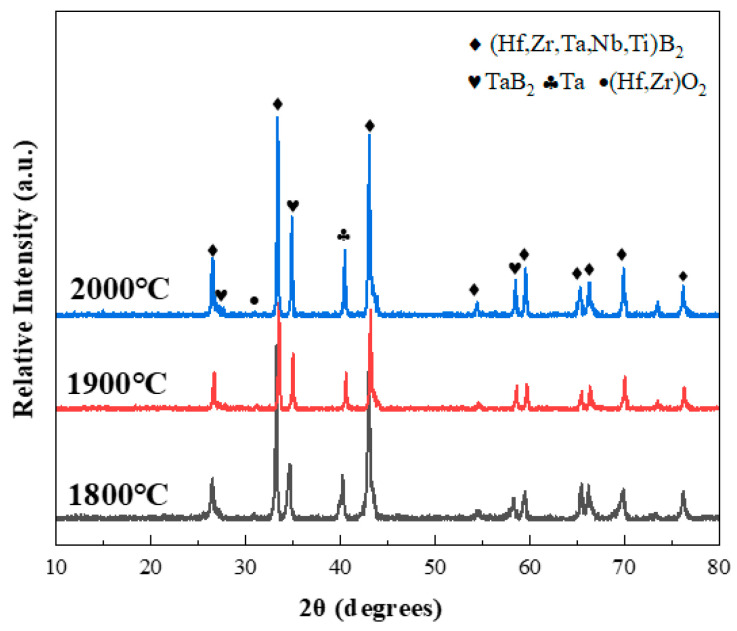
XRD patterns of composite ceramics sintered at different temperatures with 20 vol% Ta content.

Figure 1 presents the XRD patterns of the composite ceramics at various sintering temperatures with 20 vol% Ta content. As shown in the figure, as the sintering temperature increases, the diffraction peaks of the composite ceramics change only slightly, with the main peaks corresponding to (Ti,Hf,Zr,Nb,Ta)B_2_, Ta, and TaB_2_. The formation of the TaB_2_ compound results from the reaction between (Ti,Hf,Zr,Nb,Ta)B_2_ and metallic Ta. A few (Hf,Zr)O_2_ diffraction peaks are also observed, which may result from oxide contamination on the surface of the raw tantalum powder or its introduction during the ball milling process.

**Figure 2:**
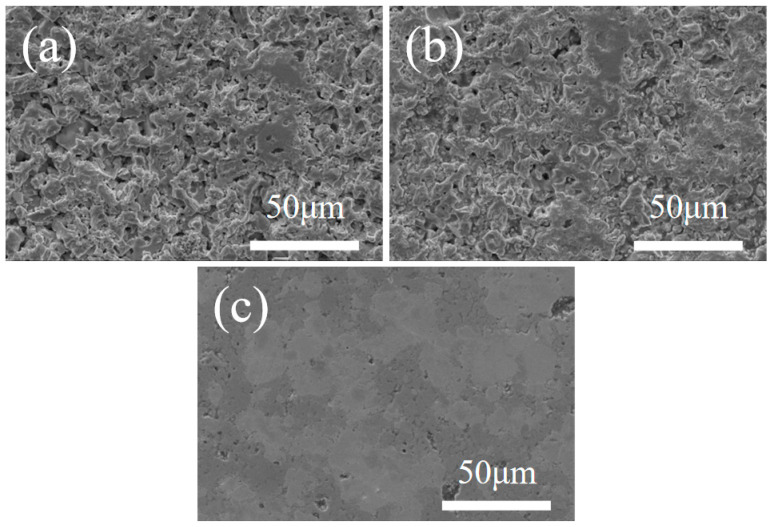
SEM micrographs of composite ceramics sintered at different temperatures with 20 vol% Ta content. (**a**) 1800 °C, (**b**) 1900 °C, and (**c**) 2000 °C.

Figure 2 presents SEM micrographs of the polished surfaces of the composite ceramics sintered at different temperatures. At a Ta content of 20 vol%, the surface pore density decreases with increasing sintering temperature (Figure 2). Figure 2a shows the polished surface of the 20 vol% Ta specimen sintered at 1800 °C. SEM observations indicate a loosely consolidated surface with abundant open pores distributed throughout the microstructure. Upon increasing the temperature to 1900 °C (Figure 2b), the pore population decreases, and the surface exhibits higher densification. Further raising the temperature to 2000 °C leaves only a few residual pores on the polished surface, indicating that higher sintering temperatures promote densification of the composite. Accordingly, a sintering temperature of 2000 °C was selected for fabricating HEB-Ta composite ceramics by SPS.

**Figure 3:**
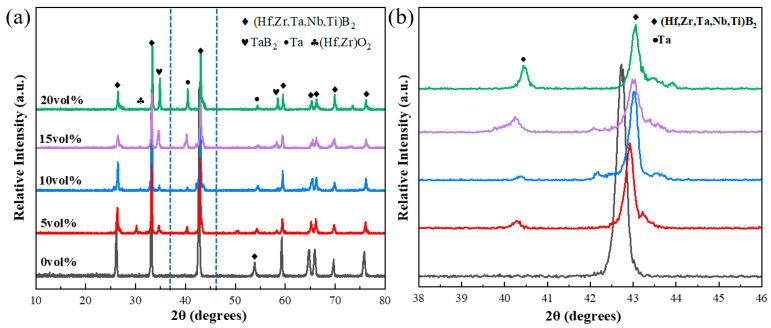
XRD patterns of the composite ceramics with Ta contents of 0, 5, 10, 15, and 20 vol%: (**a**) full 2θ range, and (**b**) enlarged region (38–46°).

Figure 3 compiles the XRD patterns recorded for composite ceramics with varied Ta contents. At 2000 °C, across all Ta contents, the principal phase is (Ti,Hf,Zr,Nb,Ta)B_2_, with Ta and TaB_2_ as secondary phases (Figure 3). The diffraction peak intensities of Ta and TaB_2_ increase monotonically with Ta content (Figure 3a). This behavior is attributable to high-temperature interactions between Ta and the (Ti,Hf,Zr,Nb,Ta)B_2_ matrix. These interactions perturb the local homogeneity of the five constituent metal ions and facilitate TaB_2_ formation. Weak diffraction peaks from oxide phases are also present, attributable to surface oxides on the metal powders introduced during ball milling.

Figure 3b shows that the principal (Ti,Hf,Zr,Nb,Ta)B_2_ diffraction peak shifts to higher angles. This shift is attributable to the smaller atomic radius of Ta (~146 pm) relative to some constituents—particularly Zr (160 pm) and Hf (159 pm)—and comparable to Ti (147 pm) and Nb (146 pm). With increasing Ta content, Ta atoms partially substitute larger-radius species (e.g., Zr or Hf), leading to a decrease in the lattice parameter and a shift in the diffraction peak toward higher angles.

**Figure 4:**
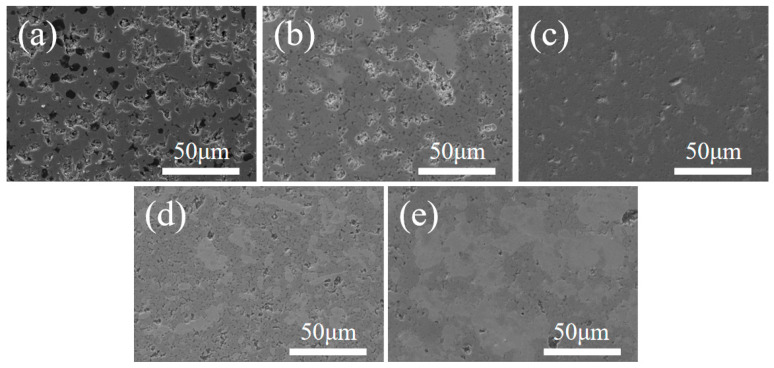
SEM pictures of polishing plane of composite ceramics with different Ta contents. (**a**) 0 vol%, (**b**) 5 vol%, (**c**) 10 vol%, (**d**) 15 vol%, and (**e**) 20 vol%.

Figure 4 presents SEM micrographs of the polished surfaces of composite ceramics with varying Ta contents. At 2000 °C, increasing Ta content reduces the surface pore population, consistent with open porosity measurements. Figure 4a shows the polished surface of monolithic (Ti,Hf,Zr,Nb,Ta)B_2_ ceramic, where numerous open pores indicate that full densification is not achieved at 2000 °C. At 5 vol% Ta (Figure 4b), surface porosity decreases; at 10 vol% Ta, pores are markedly reduced, and the microstructure becomes dense. This improvement is attributed to the high-temperature ductility of Ta, which fills intergranular gaps among (Ti,Hf,Zr,Nb,Ta)B_2_ grains, accelerates grain rearrangement, and promotes densification. In addition, reactions between Ta powder surfaces and the matrix during sintering enhance the mass transfer process. With further increases in Ta content, the composite ceramics remain dense; however, grain growth of the high-entropy boride phase is observed. At 15 vol% Ta, the grain size reaches 3.57 ± 0.25 μm, attributable to Ta-induced grain rearrangement and an enhanced mass transfer process.

**Figure 5:**
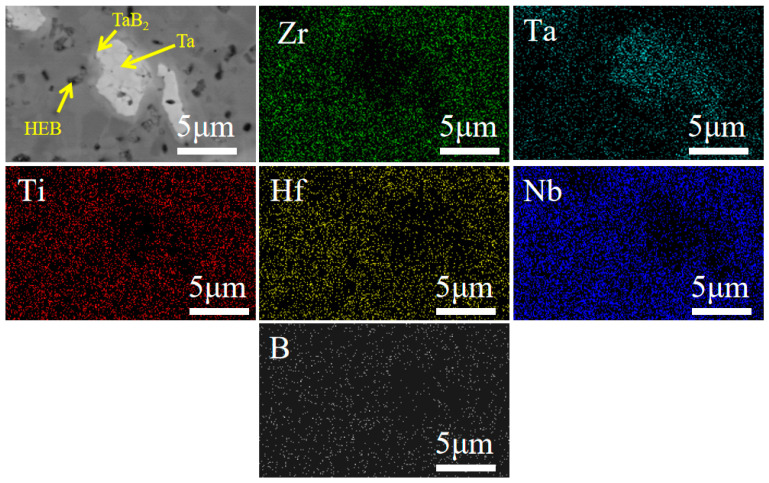
BSE-SEM image and EDS elemental maps of the polished surface of the composite ceramic containing 15 vol% Ta.

Figure 5 presents backscattered-electron SEM (BSE-SEM) micrographs and EDS elemental maps acquired from the polished surface of the composite ceramic. Under 2000 °C and 15 vol% Ta, three phase-contrast regions are apparent—white, light-gray, and dark-gray. EDS indicates that the dark-gray region contains a uniform distribution of transition-metal elements together with B and is therefore assigned to the (Ti,Hf,Zr,Nb,Ta)B_2_ matrix; this spatially homogeneous elemental distribution further corroborates the formation of a single-phase solid solution. Correlating BSE-SEM contrast with XRD/EDS signatures, the Ta-enriched white region is identified as metallic Ta, whereas the surrounding light-gray region, comprising Ta and B, is attributed to TaB_2_ formed by reaction of metallic Ta with the ceramic matrix.

**Figure 6:**
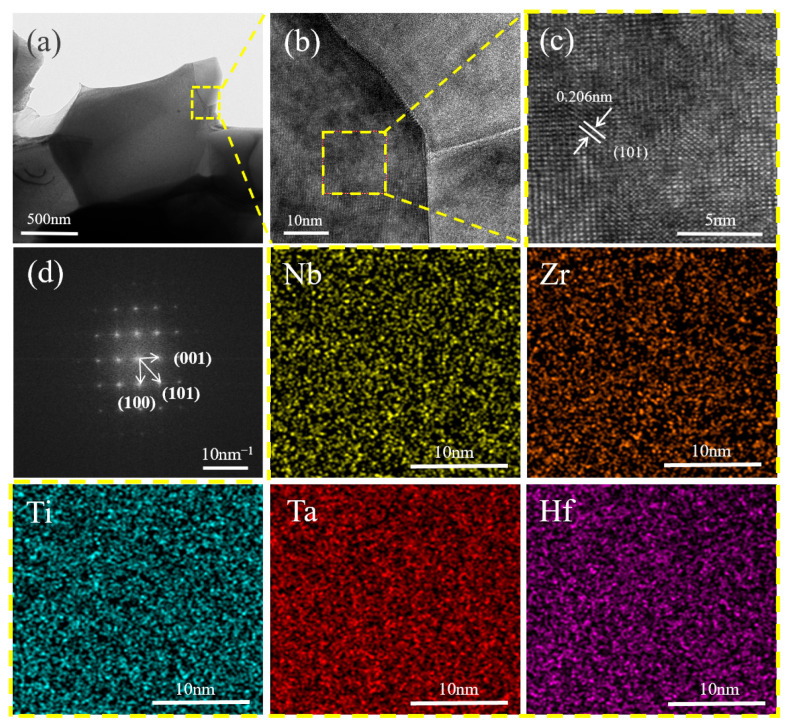
TEM characterization of the HEB matrix in the 15 vol% Ta composite ceramic: (**a**) low-magnification bright-field TEM overview, (**b**) magnified view of a grain boundary region, (**c**) HRTEM lattice fringes, and (**d**) SAED pattern.

Figure 6 summarizes TEM characterization of the HEB-Ta composite containing 15 vol% Ta. Figure 6b presents a high-resolution transmission electron microscopy (HRTEM) image of the composite, in which interfaces between HEB grains are clean, with no impurity phases at grain boundaries. In the HRTEM image of the HEB phase (Figure 6c), lattice fringes with a spacing of 0.206 nm match the (101) plane of the hexagonal HEB structure; this d-spacing (~2.06 Å) agrees with the value derived from XRD (~2.06 Å). The unit-cell parameters (a = b = 0.308 nm, c = 0.324 nm) are consistent with the XRD results, and the well-defined fringes further evidence a periodic crystal lattice. EDS mapping reveals uniform distributions of Nb, Zr, Ti, Ta, and Hf without detectable elemental enrichment or segregation, indicating that the metal atoms do not exist as an independent phase but are uniformly incorporated into the ceramic matrix, forming a single-phase solid solution. Selected-area electron diffraction (SAED) from the HEB phase (Figure 6d), together with the fast Fourier transform (FFT) of the HRTEM image, exhibits sharp, symmetric diffraction spots that confirm long-range order and hexagonal symmetry, consistent with XRD.

**Figure 7:**
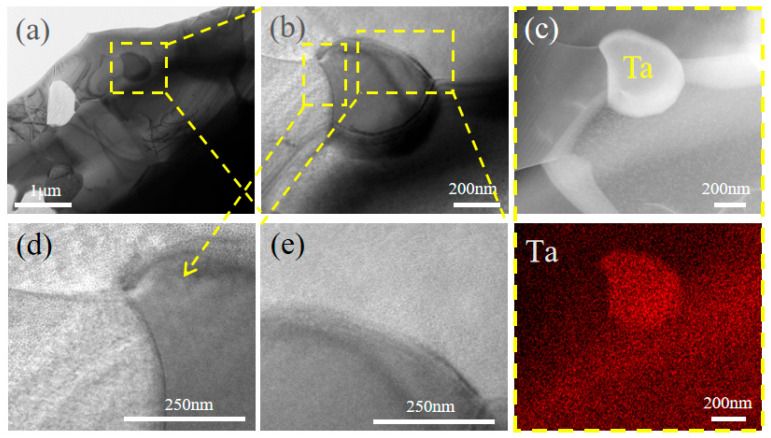
TEM characterization of the 15 vol% Ta composite ceramic: (**a**) low-magnification bright-field TEM overview, (**b**) enlarged view of a metallic Ta inclusion, (**c**) magnified view of the metallic Ta, and (**d**,**e**) magnified views of the interfaces surrounding the metallic Ta.

Figure 7 presents TEM images and EDS elemental maps of the HEB-Ta composite ceramic. Figure 7a shows that metallic Ta retains its morphology and is well bonded to the ceramic matrix. The interface between the Ta-containing region and the surrounding high-entropy boride (HEB) matrix is free of impurities or secondary-phase accumulation, exhibiting a sharp, well-defined boundary. Figure 7c shows a dark-field TEM image of the composite. EDS mapping reveals a Ta-enriched (red) region whose spatial distribution coincides with the morphology observed by TEM; combined with crystal structure analysis, this region is identified as metallic Ta. Figure 7d,e show magnified views of the Ta/HEB interface. The Ta-rich region remains discrete with limited intermixing of other elements, and the interface is clean. These observations indicate that Ta persists at interfaces and within the microstructure as a discrete metallic phase, with no evidence of extensive interfacial reactions under the processing conditions. The addition of Ta does not adversely affect the HEB phase: elements in the HEB ceramic remain uniformly distributed, and the phase maintains hexagonal symmetry and single-phase solid-solution character.

Table 1 summarizes the open porosity, density, hardness, fracture toughness, and grain size of composite ceramics with varying Ta contents. At 2000 °C, the open porosity exhibits a non-monotonic dependence on Ta content, decreasing initially and increasing thereafter. At 10 vol% Ta, the open porosity reaches a minimum of 0.15%, indicating that Ta addition enhances the sinterability of the composite. The bulk density increases with Ta content, consistent with the higher density of Ta (16.6 g·cm^−3^) relative to the high-entropy boride matrix.

At 2000 °C, the hardness exhibits a non-monotonic dependence on Ta content—initially increasing and subsequently decreasing. The maximum hardness (20.79 ± 0.17 GPa) occurs at 15 vol% Ta; this maximum is associated with reduced open porosity, increased bulk density, and grain refinement. Increasing the Ta content to 20 vol% reduces the hardness to 16.22 ± 0.11 GPa, owing to an increased volume fraction of the softer metallic Ta phase (hardness 240–393 MPa) and grain growth.

With increasing Ta content, the fracture toughness of the composite ceramic increases initially and then decreases. At 15 vol% Ta, the fracture toughness reaches a maximum of 4.31 ± 0.12 MPa·m^1/2^, attributable to the superior plastic deformability of metallic Ta. The fracture surface is rough and exhibits a mixed transgranular-intergranular mode, which increases crack propagation energy and thereby enhances toughness. Owing to grain coarsening, which weakens the material’s toughness, the fracture toughness of HEB-20Ta (20 vol% Ta) decreases to 3.67 ± 0.26 MPa·m^1/2^.

**Figure 8:**
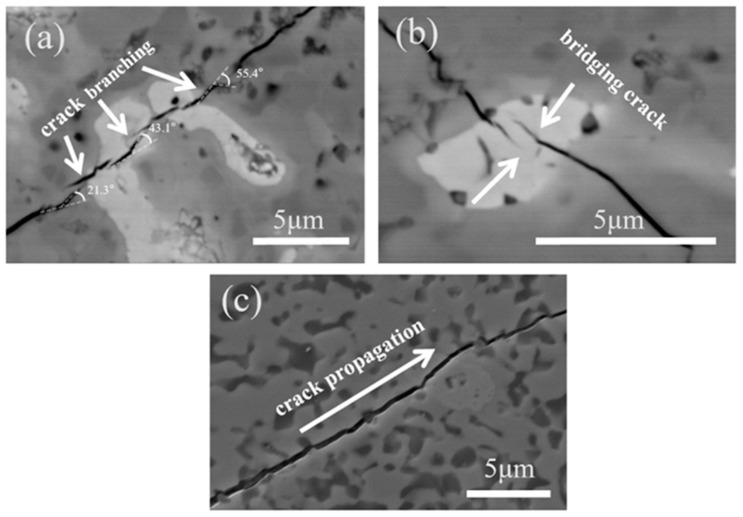
SEM micrographs of planar crack propagation on the polished surface of the 15 vol% Ta composite ceramic. (**a**) crack deflection in Ta-containing regions; (**b**) crack bridging in Ta-containing regions; (**c**) crack propagation path in Ta-free regions.

Figure 8 presents SEM and BSE-SEM images of crack paths on the polished surface of the composite ceramic. Figure 8a,b show BSE-SEM magnifications of crack propagation in Ta-containing regions, whereas Figure 8c shows an SEM image of a Ta-free region. When the propagating crack encounters metallic Ta, numerous instances of crack deflection, branching, and bridging are observed. By contrast, the crack in Figure 8c propagates almost in a straight line, with no obvious deflection, traversing the entire micrograph. In Figure 8a,b, crack branching and bridging occur primarily within the light-gray regions; correlation with Figure 5 indicates that these light-gray regions correspond to the metallic Ta phase. Because metallic Ta deforms ductilely, the resulting crack bridging and branching dissipate crack propagation energy [48], indicating that Ta incorporation contributes to toughening.

**Figure 9:**
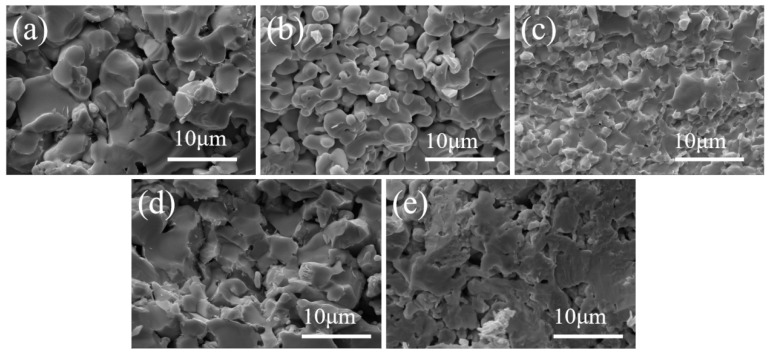
SEM images of the fracture surface of the composite ceramics with different Ta contents: (**a**) 0 vol%, (**b**) 5 vol%, (**c**) 10 vol%, (**d**) 15 vol%, and (**e**) 20 vol%.

Figure 9 presents SEM micrographs of fracture surfaces of composite ceramics with varying Ta contents. With increasing Ta content, the apparent porosity decreases markedly. The HEB grain size shows a trend of decreasing initially and then increasing; at 10 vol% Ta, the HEB grain size reaches a minimum of 2.33 μm. This suggests that metallic Ta exerts a grain boundary pinning effect that suppresses grain growth and promotes grain refinement.

At 5–15 vol% Ta, the fracture surfaces are rough and exhibit a mixed transgranular-intergranular mode (Figure 9c,d). At 20 vol% Ta, the fracture surface becomes smoother and is dominated by transgranular fracture, consistent with characterization results showing decreased fracture toughness.

**Figure 10:**
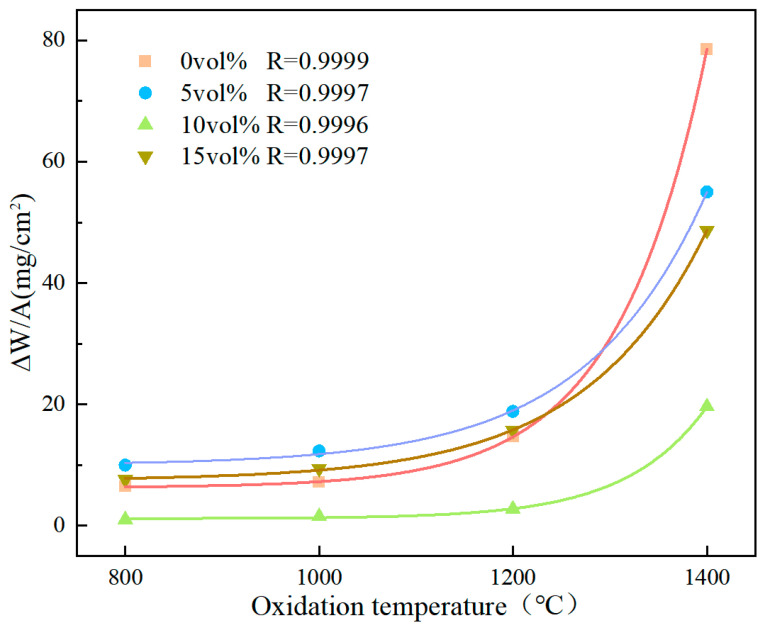
Oxidation-induced mass gain of Ta-containing composite ceramics as a function of oxidation temperature and Ta content.

Figure 10 presents oxidation mass-gain curves for composite ceramics with varying Ta contents at different temperatures. With increasing oxidation temperature, the mass gain increases. When the temperature exceeds 1200 °C, the oxidation mass gain of the composite ceramics increases markedly—particularly for compositions without metallic Ta—indicating poorer high-temperature stability and oxidation resistance. The curves exhibit a parabolic trend consistent with typical oxidation kinetics, and the coefficient of determination for each curve is R^2^ ≥ 0.9996.

With increasing Ta content, the oxidation mass gain exhibits a non-monotonic trend—initially decreasing and then increasing. At 10 vol% Ta, the oxidation mass gain reaches a minimum. This is related to the highest bulk density of the composite ceramics at this composition. Simultaneously, Ta preferentially forms Ta_2_O_5_ during oxidation, generating a dense protective oxide layer that impedes further oxygen diffusion and reduces the contact between the ceramic matrix and oxygen.

However, as the Ta content continues to increase, the oxidation mass gain of the composite ceramics gradually increases. This is attributed to the atomic radius difference between Ta and other elements in the ceramics, with the addition of a large amount of Ta disrupting lattice regularity and inducing lattice distortion in the high-entropy boride phase. This induces lattice defects [49]. These defects provide pathways for oxygen diffusion, facilitating its penetration into the ceramic interior and accelerating the oxidation reaction, thereby increasing the oxidation mass gain for the HEB-15Ta.

**Figure 11:**
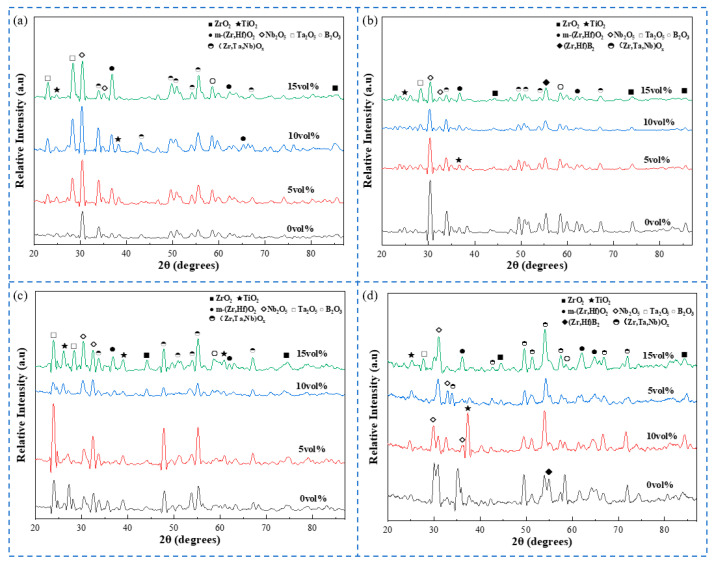
XRD patterns of composite ceramics with different Ta contents after oxidation at different temperatures: (**a**) 800 °C, (**b**) 1000 °C, (**c**) 1200 °C, and (**d**) 1400 °C.

Figure 11 presents the XRD patterns of composite ceramics with varying Ta contents after oxidation at different temperatures. With increasing oxidation temperature, the diffraction peak intensities of the oxides increase significantly. At 800 °C, the number of oxide phases is limited, and the diffraction peaks are sharp but weak, indicating that the oxidation reaction is limited at this temperature. The phase crystals are ordered but present in small quantities.

At oxidation temperatures between 1000 and 1200 °C, the diffraction peak intensities increase overall, the number of phase types increases, and more solid solution phases such as Ta and Nb are formed. This suggests that higher temperatures promote a more complete oxidation reaction, leading to the formation of additional phases and an increase in crystallinity. At 1400 °C, the diffraction peaks tend to broaden, due to enhanced grain growth and intensified diffusion reactions between phases at high temperature. Although the phase types remain consistent with those at medium temperatures, the crystalline state changes due to high-temperature kinetics.

At an oxidation temperature of 1400 °C, the primary phases in the composite ceramics include m-(Zr, Hf)O_2_, (Zr, Ta, Nb)O_x_, (Zr, Hf)B_2_, B_2_O_3_, Ta_2_O_5_, and others. As Ta content increases, the diffraction peak intensities of m-(Zr, Hf)O_2_ and (Zr, Ta, Nb)O_x_ solid solutions increase, while the diffraction peak intensities of other oxides decrease. This suggests that Ta doping promotes the formation of oxide solid solutions and inhibits the formation of certain oxide phases. Diffraction peaks of B_2_O_3_ were observed in all composite ceramics, with peak intensity decreasing as Ta content increased. The diffraction peaks of Ta_2_O_5_, formed upon oxidation, gradually increased due to excessive Ta oxidation, which led to Ta_2_O_5_ formation. Ta_2_O_5_ dissolves into the B_2_O_3_ phase. As Ta content increases to 15 vol%, the diffraction peak intensities of all oxides gradually increase, and the shape of some oxide peaks changes from sharp to broadened. Excessive Ta may introduce lattice defects, accelerating the diffusion of oxygen and thereby reducing oxidation resistance. At 10 vol% Ta, the HEB peak of the composite ceramic is the most intense and sharp, indicating optimal high-temperature stability. This is due to the fact that the addition of Ta and other elements effectively enhances the oxidation resistance of the composite ceramics [29], which is also related to their highest density.

**Figure 12:**
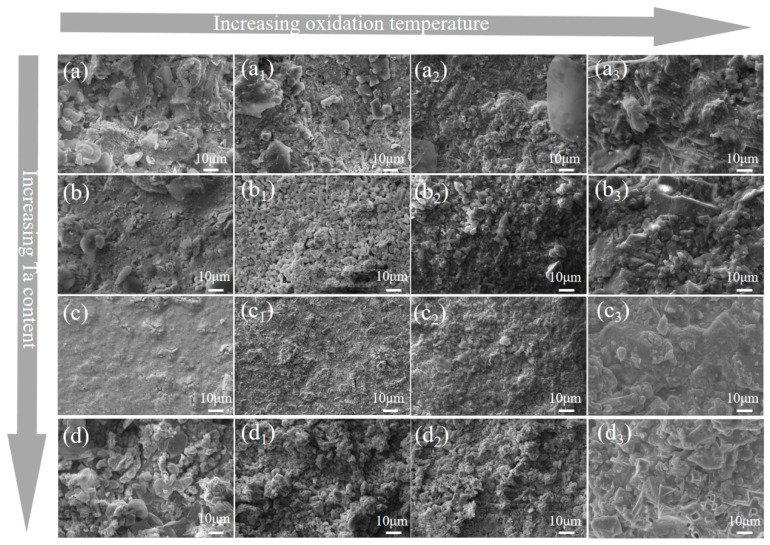
Multiphase ceramic oxide layer plane picture: (**a**) 0 vol%, (**b**) 5 vol%, (**c**) 10 vol%, and (**d**) 15 vol%. The oxidation temperature was 800 °C without numbering, and the oxidation temperature was 1000 °C, 1200 °C and 1400 °C with small numbers 1, 2 and 3, respectively. (**a**) 0 vol%, 800 °C; (**a_1_**) 0 vol%, 1000 °C; (**a_2_**) 0 vol%, 1200 °C; (**a_3_**) 0 vol%, 1400 °C. (**b**) 5 vol%, 800 °C; (**b_1_**) 5 vol%, 1000 °C; (**b_2_**) 5 vol%, 1200 °C; (**b_3_**) 5 vol%, 1400 °C. (**c**) 10 vol%, 800 °C; (**c_1_**) 10 vol%, 1000 °C; (**c_2_**) 10 vol%, 1200 °C; (**c_3_**) 10 vol%, 1400 °C. (**d**) 15 vol%, 800 °C; (**d_1_**) 15 vol%, 1000 °C; (**d_2_**) 15 vol%, 1200 °C; (**d_3_**) 15 vol %, 1400 °C.

Figure 12 presents the SEM image of the composite ceramic oxide layer. As the oxidation temperature increases, the grain size of the composite ceramic oxide layer increases, and the surface becomes increasingly uneven, particularly with varying Ta content. This is due to the escape of volatile gaseous products, such as B_2_O_3_ and CO, during oxidation, which results in a rough surface. At 10 vol% Ta, the surface of the oxide layer is relatively smooth. This is because Ta reacts with oxygen to form more Ta_2_O_5_, which dissolves in the B_2_O_3_ glass phase, increasing viscosity and reducing the oxygen diffusion rate [50,51]. The glass phase fills the pores and defects on the oxide layer’s surface, forming a dense oxide layer that prevents further oxygen ingress and enhances the oxidation resistance of the composite ceramics.

**Figure 13:**
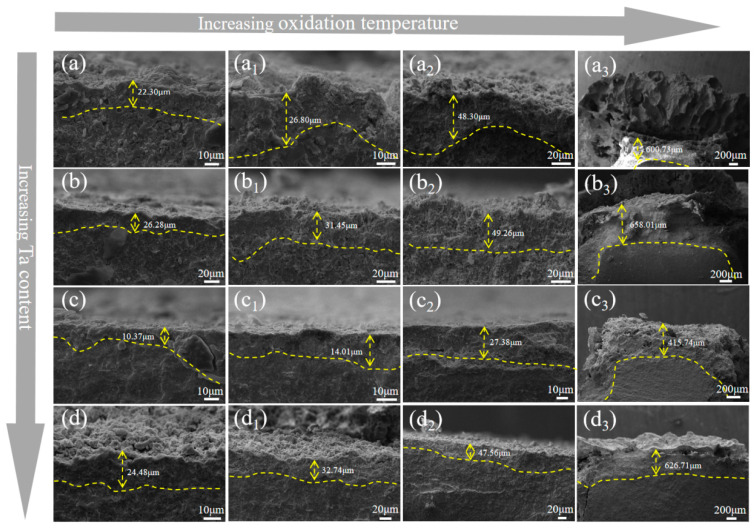
SEM cross-sectional images of the oxide layer of the multiphase ceramic: (**a**) 0 vol%, (**b**) 5 vol%, (**c**) 10 vol%, and (**d**) 15 vol%. Oxidation at 800 °C is shown without a numeric label; oxidation at 1000, 1200, and 1400 °C is indicated by the small numbers 1, 2, and 3, respectively. (**a**) 0 vol %, 800 °C; (**a_1_**) 0 vol%, 1000 °C; (**a_2_**) 0 vol%, 1200 °C; (**a_3_**) 0 vol%, 1400 °C. (**b**) 5 vol%, 800 °C; (**b_1_**) 5 vol%, 1000 °C; (**b_2_**) 5 vol%, 1200 °C; (**b_3_**) 5 vol%, 1400 °C. (**c**) 10 vol%, 800 °C; (**c_1_**) 10 vol%, 1000 °C; (**c_2_**) 10 vol%, 1200 °C; (**c_3_**) 10 e, 1400 °C. (**d**) 15 vol%, 800 °C; (**d_1_**) 15 vol%, 1000 °C; (**d_2_**) 15 vol%, 1200 °C; (**d_3_**) 15 vol%, 1400 °C.

Figure 13 presents the cross-sectional image of the composite ceramic oxide layer. As the oxidation temperature increases, the oxidation depth of each sample group gradually increases. Between 800 °C and 1200 °C, the oxidation of each sample group is relatively mild, with thinner oxide layers that show only a slight increase in thickness. At 1400 °C, the oxide layer of each sample group adopts a typical sponge-like structure. This phenomenon is attributed to the reaction between oxygen, boron, and carbon elements, forming B_2_O_3_ and CO gases. These gases escape from the composite ceramics, resulting in the formation of a sponge-like pore structure [52]. At 1400 °C, the sample group without Ta exhibits a thicker oxide layer, with a measured thickness of 600.73 μm, accompanied by a thicker sponge-like structure. This indicates that the oxidation reaction in this sample group is intense, and the large grains or discontinuities formed at high temperatures provide enhanced volatilization pathways for B_2_O_3_, exacerbating the porosity of the oxide layer. It can also be inferred that the oxidation resistance of this sample group is relatively poor.

With increasing Ta content, the oxide layer thickness at 5 vol% and 15 vol% Ta is slightly higher than that of the Ta-free group, due to the lower density and higher porosity of the 5 vol% group. Oxygen reacts with the ceramic powder and Ta, resulting in increased oxidation and oxide formation, forming a thicker oxide layer compared to the Ta-free group. At 15 vol% Ta, excessive Ta in the composite ceramics reacts with oxygen, forming a thicker oxide layer. However, the HEB-10Ta group, with its high density and sponge-like structure, which prevents gas escape, exhibits the lowest oxidation weight gain and the thinnest oxide layer compared to other groups. At 800 °C, the oxide layer thickness is only 10.37 μm, and at 1400 °C, it is 415.74 μm. This is primarily due to Ta addition, which significantly increases the density of the composite ceramics, hindering the effective ingress of external oxygen and thereby reducing the oxidation degree.

## 4. Conclusions

In this study, HEB and Ta powders were uniformly mixed, and dense HEB–Ta composite ceramics were fabricated via SPS. The influence of varying Ta contents on the phase composition, microstructure, mechanical properties, and oxidation resistance of the composite ceramics was systematically investigated. The following key conclusions can be drawn:

Dense HEB-Ta composite ceramics were successfully fabricated via SPS at a heating rate of 100 °C/min, under 2000 °C and 30 MPa, with a 10 min hold time. Metallic Ta was well preserved and uniformly distributed in the composite ceramic matrix. At high temperature, a small amount of Ta reacted with the ceramic powder to form TaB_2_. As the Ta content increased, the open porosity initially decreased and then stabilized. At 10 vol% Ta, the open porosity of the composite ceramics reached its lowest value, 0.15%. As the Ta content increased, the average grain size of the composite ceramics first decreased and then increased. At 10 vol% Ta, the composite ceramics exhibited the smallest grain size, measuring 2.33 ± 0.14 μm. The hardness of the composite ceramics first increased and then decreased. At 15 vol% Ta, the composite ceramics exhibited the highest hardness and fracture toughness, measuring 20.79 ± 0.17 GPa and 4.31 ± 0.12 MPa·m^1/2^, respectively. At 20 vol% Ta, the hardness and fracture toughness decreased slightly, due to the increased presence of the softer metallic Ta phase and grain growth in the composite ceramics.

As the oxidation temperature increased, both the oxidation mass gain and the oxide layer thickness of each composite ceramic group showed an increasing trend. Between 1200 °C and 1400 °C, the oxidation mass gain and oxide layer thickness exhibited a parabolic variation. The main phases after oxidation included m-(Zr,Hf)O_2_, (Zr,Ta,Nb)O_x_, (Zr,Hf)B_2_, B_2_O_3_, and Ta_2_O_5_. At 5 vol% and 15 vol% Ta, the oxidation mass gain and oxide layer thickness were greater than those at 10 vol% Ta, because the 5 vol% group had lower density and higher porosity, facilitating oxygen ingress and reaction with the composite ceramics. At 15 vol% Ta, the higher Ta content led to more Ta reacting with oxygen, resulting in a thicker oxide layer and a larger oxidation mass gain. At 10 vol% Ta, the composite ceramics exhibited the lowest oxidation mass gain and oxide layer thickness; at 800 °C, the oxidation mass gain was 6.48 mg cm^−2^, and the oxidation depth was only 10.37 μm. This was because adding an optimal amount of Ta increased the density of the composite ceramics, making external oxygen penetration more difficult. Simultaneously, Ta reacted with oxygen to form Ta_2_O_5_, which dissolved in the B_2_O_3_ glassy phase, thereby increasing viscosity and reducing the oxygen diffusion rate. The synergy of these factors optimized the oxidation resistance of the composite ceramics.

## Figures and Tables

**Table 1:** Density, grain size and mechanical properties of composite ceramics with different Ta content.

Group	Open Porosity (%)	Density (g/cm^3^)	Grain Size of HEB (μm)	Hardness (GPa)	Fracture Toughness (MPa·m^1/2^)
HEB-0Ta	7.28	7.55	7.85 ± 0.09	17.02 ± 0.24	3.17 ± 0.15
HEB-5Ta	4.40	8.27	4.37 ± 0.10	18.37 ± 0.31	3.94 ± 0.21
HEB-10Ta	0.15	9.04	2.33 ± 0.14	20.63 ± 0.22	4.24 ± 0.13
HEB-15Ta	0.43	9.42	3.57 ± 0.25	20.79 ± 0.17	4.31 ± 0.12
HEB-20Ta	0.22	9.87	5.43 ± 0.12	16.22 ± 0.11	3.67 ± 0.26

## Data Availability

All data generated or analyzed in this study are included in this article.
